# Quality-Driven Design of Pandan-Flavored Sponge Cake: Unraveling the Role of Thermal Processing on Typical Pandan Aroma

**DOI:** 10.3390/foods13193074

**Published:** 2024-09-26

**Authors:** Xiao Chen, Ying Cao, Weijie Lan, Zixuan Gu, Wenjia He, Jianfei He, Liyan Zhao

**Affiliations:** 1 Sanya Institute of Nanjing Agricultural University, Sanya 572024, China; xiaochen@njau.edu.cn (X.C.); weijie.lan@njau.edu.cn (W.L.); 2 College of Food Science and Technology, Nanjing Agricultural University, Nanjing 210095, China; 3 Department of Plant Sciences, North Dakota State University, Fargo, ND 58108, USA; zixuan.gu@ndsu.edu; 4 College of Food Science and Technology, Zhejiang University of Technology, Hangzhou 310014, China; wenjhe@zjut.edu.cn (W.H.); jfhe@zjut.edu.cn (J.H.)

**Keywords:** volatile compounds, GC-IMS, GC-O-MS, texture, odor-taste interaction

## Abstract

Pandan (*Pandanus amaryllifolius* Roxb.) has been used in the production of bakery goods either as a functional ingredient or a natural flavoring that, when roasted, exerts a fragrant rice-like aroma and an attractive green color. This study elucidated the typical aroma compounds from pandan leaves and explored the influence of thermal treatments on their aroma profiles using GC-O-MS, E-nose, and GC-IMS analyses. The effects of formulation and baking conditions on the qualities of pandan-flavored sponge cake were comprehensively evaluated through a holistic approach covering several aspects including cake batter gravity, color, texture, and sensory characteristics. The baking treatment introduced more types of pleasant aromas (9 aromas vs. 17 aromas) and increased the odor intensities of the original volatile compounds, especially for the roasted and steamed rice-like odors. The increased amount of pandan flavoring reshaped the color of the cake crumb (especially for the L* and a* spaces) and significantly decreased the hardness (3.87 N to 1.01 N), gumminess (3.81 N to 0.67 N), and chewiness (13.22 mJ to 4.56 mJ) of the sponge cake. The perceived intensities of bitterness and sweetness can be adjusted by modulating the levels of 2-phenylethanol, 2-methyl-1-butanol, hexyl alcohol, and decanal, along with the total alcohols and aldehydes, due to their significant correlations revealed by correlation heatmap analyses.

## 1. Introduction

*Pandanus amaryllifolius* Roxb., commonly known as pandan, is mainly distributed in tropical regions, belonging to *Pandanaceae* in the screw pine family. Pandan is native to Indonesia and has spread to many South and Southeastern Asian countries, such as India, Malaysia, Thailand, and China. Historically, pandan leaves were traded inside the Indonesian Archipelago and shipped to India and China along with other common species, i.e., nutmegs, candlenuts, and cloves. Currently, the global annual production of fresh pandan leaves exceeds four million tons, and Asia accounts for > 80% of this production with Indonesia and Thailand as the major producers. Pandan leaves have been medicinally used to reduce fever, relieve indigestion, and refresh the body, and the oil produced from the leaves is believed to have antispasmodic and stimulant properties, effective against epilepsy, rheumatism, headaches, and for skin disease [1,2,3]. As crafted by the Peranakan Chinese, pandan flavoring, resembling sweet and delightful aromas, has become part of their cultural identity and gained popularity globally for a variety of food production, including sweets, baked goods, and home cooking. Overall, pandan leaves hold important social and economic significance thanks to their versatile applications, unique characteristics, high added values, and significant production potential.

Diverse types of phytochemical compounds have been detected in pandan leaves, including flavonoids, tannins, saponin, anthraquinone glycoside, cardiac glycoside, alkaloids, proteins, terpenoids, and starches [1,2]. The aroma of *P. amaryllifolius* leaves is unique and characterized by nutty and pleasant attributes, which are of commercial interest in flavor industries for the production of pandan-flavored food items. Early investigations have suggested that 2-acetyl-1-pyrroline (2AP) is the primary aroma compound of pandan leaves, although volatile esters, ketones, aldehydes, and alcohols can also be detected [4,5,6]. 2AP shows commercial significance, imparting the typical aroma of cooked fragrant rice varieties, such as basmati rice in India and Thai jasmine rice. Thus, pandan leaves have been traditionally used with non-aromatic rice varieties to achieve a basmati-like aroma in cooked rice [7]. The leaves can be used to season vegetables or meat products, or mixed with other flavor-enhancing sauces. The unique aroma also promotes the harvesting of *P. amaryllifolius* leaves for the perfume industry, and numerous well-known brands now create ethnic Asian herbal perfumes, including pandan fragrances [8,9]. Notably, the aromatic ingredients of pandan leaves, particularly 2AP, are highly unstable and easily diffuse into the air, thus pandan-derived food products lose their signature scent under inappropriate thermal processing conditions. 

Nowadays, with the increased health consciousness of consumers, plant-based natural colorings have gained rising popularity. The abundance of chlorophyll and safety advantages have made pandan leaves an ideal natural colorant in the production of various foodstuffs, such as pandan-flavored custards, ice cream, bakery items, and others [10]. Sponge cake is a particularly popular bakery product presenting a complex structure and chemical composition. During baking, diverse ingredients (i.e., salt, flour, fat, egg, and sugar) undergo several reaction pathways, leading to an aerated and light structure with numerous volatiles, which is associated with the typical flavor. Notably, the selection of raw materials and the alterations in the production process can modify aroma composition and cake structure. For example, Papageorgiou et al. [11] found that a cocoa cake with 30% carob flour received the highest score for overall acceptability; and Huang and Yang [12] reported that *Eucheuma* powder increased the gravity, viscoelasticity, and viscosity of cake batters. Although pandan leaves have been used as a natural colorant for sponge cake, the aspects of pandan replacement in terms of cake quality attributes and sensory characteristics have not been assessed in the literature.

Therefore, this paper aims to elucidate the potential typical aroma compounds in pandan leaves and explore how these aroma compounds change upon thermal treatments. Additionally, the effects of baking conditions and formulation on the qualities of pandan-flavored sponge cake and its optimization are also performed in this study. A holistic approach covering several aspects, including cake batter gravity, color, texture, and sensory characteristics, is used to understand this underlying phenomenon. The research results provide useful insights into developing and utilizing pandan leaves while retaining their typical aromas, which are important for increasing their added value, enriching product diversity, and optimizing high-value processing technologies for pandan leaves. 

## 2. Materials and Methods

### 2.1. Pandan Leaf Juice Preparation

Fresh pandan leaves were purchased from a local market (Wanning, China) in March 2024. The pandan leaves (approximately 250 g) were cut into small pieces after removing the yellow parts and roots and were soaked in water for 30 min. Then one-third of the leaves were put into a wall-breaker (L12-P726 wall-breaker, Jiuyang Co., Ltd., Jinan, China) with 250 g of drinking water (Wahaha Group Co., Ltd., Hangzhou, China) and mixed thoroughly. The remaining leaves were then added into the wall-breaker and thoroughly pulverized, yielding pandan leave homogenate. The homogenate was poured out and filtered through a 100-mesh sieve to obtain the pandan leaf juice. The residue was put into a 16-mesh gauze to squeeze out the juice and then combined with the previous fraction. The pandan juice was stored in a 500 mL sealed jar (Xuzhou Zhuoyang Glass Products Co., Ltd., Xuzhou, China) and kept refrigerated (4 °C) until required.

### 2.2. Pandan-Flavored Cake Elaboration

The traditional sponge cake formula was adapted from Kazanci, et al. [13] with slight modifications for the current study. Table 1 shows the formula of the pandan-flavored cake containing four pandan juice levels. Corn oil and butter were mixed and heated to 60 °C in a water bath (SC-5A, Nanjing Xian’ou Instrument Manufacturing Co., Ltd., Nanjing, China). Low gluten flour, corn starch, and egg yolk (separated from the whole egg) were added into the emulsified oil and then stirred in the same direction for a while. Different proportions of pandan leaf juice (8.4, 11.2, 14, or 16.8 g) were added to the egg yolk batter, representing the 10.3%, 13.3%, 16.1%, and 18.8% supplement of pandan flavorings into the sponge cake. Subsequently, the egg white was poured into a bowl; the sugar and salt mixtures were then added and mixed with a whisk (D301, Hefei Rongshida Habitat Technology Co., Ltd., Hefei, China) on a speed 4 setting for 3 min. The whipped egg white was added to the pandan-flavored egg yolk batter and then sufficiently mixed by hand with a plastic scraper until smooth. 

The prepared cake batter was poured into a mold (length 10 cm × width 8 cm × height 5 cm) and placed in a preheated oven (PT2531, Midea Group Co., Ltd., Foshan, China) at 130/140/150 °C for 45 min. Before removing from the pans, the cakes were allowed to cool at room temperature for 2 h. The cake samples were packed into polypropylene bags before sensory evaluation and physicochemical analysis. The pandan-flavored cake samples supplemented with 0%, 10.3%, 13.3%, 16.1%, and 18.8% pandan leaf juice were designated as the control, PFC-1, PFC-2, PFC-3, and PFC-4, respectively. At least eight cake samples were prepared for each treatment and all treatments were randomly performed.

### 2.3. Specific Gravity of the Cake Batter

As reported by Rodríguez-García et al. [14], the batter density was determined as the ratio between the mass of the cake batter and the mass of the same volume of distilled water. The total weight of the specific gravity cup (90 mL, UBS Trading Co. Ltd., Chaozhou, China) filled with distilled water was recorded as m_1_. The pandan-flavored cake batter (containing various percentages of pandan juice) was transferred to the same gravity cup and the total weight was recorded as m_2_. The batter density was calculated using the following Equation (1):

(1) $$\mathrm{Relative}\;\mathrm{batter}\;\mathrm{density}=\frac{m_{2}-m_{0}}{m_{1}-m_{0}}$$

where $m_{2}$ is the total mass of the specific gravity cup plus cake batter, $m_{1}$ is the total mass of the specific gravity cup plus distilled water, and $m_{0}$ is the mass of the empty gravity cup.

### 2.4. Colorimetric Measurement

The color of the top, middle, and bottom of the cake samples was measured by a colorimeter (NR10QC, Shenzhen SUNSHI Technology Co., Shenzhen, China) for yielding L* (lightness), a* (red-green), and b* (yellow-blue) parameters. The color difference was calculated following Equation (2):

(2) $$\Delta E=\sqrt{{(∆L}^{*}{)}^{2}+{{(∆a}^{*})}^{2}+{{(∆b}^{*})}^{2}}$$

where ${∆L}^{*}$ refers to the difference in brightness between the sample and the reference (control); ${∆a}^{*}$ denotes the red/green difference between the cake sample and the reference (control); ${∆b}^{*}$ represents the difference in yellow/blue between the pandan-flavored cake and the control. 

### 2.5. Texture Evaluation

For texture analyses, pieces of crumb (1 cm × 1 cm × 1 cm) were cut from the top left, top right, bottom left, bottom right, and the center of the cake sample. The cake crumbs were immediately compressed using a TA-XT2 texture analyzer (Stable Micro Systems, Surrey, UK). The measuring conditions were as follows: cylindrical probe, TA10; force bar element size, 50 N; deformation 40%; detection speed, 60 mm/min; and starting force, 0.1 N. The parameters of hardness, adhesiveness, springiness, cohesiveness, chewiness, and gumminess were also measured. 

### 2.6. Effects of Thermal Treatments on Volatile Compounds of Pandan Leaf Juices

#### 2.6.1. Electronic Nose (E-Nose) Analysis

Fresh pandan leaf juice samples (8 g) were transferred into a 20 mL headspace vial and thermal-treated in a DHG-9123A oven (Shanghai Jinghong Experimental Equipment Co., Shanghai, China) under 80 °C, 100 °C, 120 °C, and 140 °C for 1 h, and the obtained samples were abbreviated as TPJ-80, TPJ-100, TPJ-120, and TPJ-140, respectively. The volatile profile was then analyzed by a PEN3 *E*-nose apparatus supplied by Airsense Analytics GmbH (Schwerin, Germany). The test conditions were as reported by our previous studies with no modifications [15].

#### 2.6.2. Gas Chromatography-Ion Mobility Spectrometry (GC-IMS) Analysis

The volatile compounds of TPJ-80, TPJ-100, TPJ-120, and TPJ-140 samples were analyzed by GC-IMS (FlavourSpec, G.A.S., Dortmund, Germany) under slightly modified conditions as reported by Chen et al. [15]. Briefly, an aliquot of the pandan juice sample (3 g) was placed into the headspace vial, which was immediately incubated for 15 min at 50 °C with an oscillatory at 250 rpm. Subsequently, a syringe was automatically injected into the headspace at 50 °C for balanced gas adsorption. The gas (500 μL) was injected into a nonpolar capillary FS-SE-54-CB-1 column (15 m × 0.53 mm ID × 1 μm FT) supplied by Hai Neng Science Instrument Co., Ltd. (Jinan, Shandong, China) under a splitless mode at 60 °C for 20 min. Pure nitrogen (>99.999%) was used as the drift and carrier gas. The flow rate was set as follows: initially 2.0 mL/min for 2 min, gradually increased to 10 mL/min for 10 min, and ultimately increased to 150 mL/min for 30 min. The ionization was in positive mode and triggered by a 300–500 MBq *H* ionization source. A drift tube (9.8 cm in length) was used to accommodate the resultant ions under a constant voltage of 5 kV and 45 °C. Each spectrum was scanned an average of 12 times and the flow rate of drift gas was 150 mL/min.

A series of C_4_–C_9_ ketones (Sinopharm Chemical Reagent Co., Ltd., Beijing, China) were used as external references for analyte identification by calculating retention indices (RIs). Drift time comparison was adopted as another approach for compound identification. The subsequent statistical analyses involved the following software packages, i.e., Gallery Plot Plug-in, Laboratory Analytical Viewer, GC-IMS Library Search, and Reporter Plug-in. The specific functions of the above software have been described in our previous study [15].

#### 2.6.3. Gas Chromatography-Olfactometry-Mass Spectrometry (GC-O-MS) Analysis

Fresh pandan leaf juice (control) was statically thermal-treated in an oven under 130 °C for 2 h and then cooled to room temperature for analysis (marked as TPJ-130). The control or TPJ-130 sample was placed into a 20 mL amber glass screw-capped headspace vial (ANPEL Laboratory Technologies Co., Ltd. (Shanghai, China). Each vial was incubated in a water bath at 50 °C for 20 min, followed by the volatile compound collection using the headspace solid-phase microextraction (HS-SPME) technique with a Supelco Inc. DVB/CAR/PDMS fiber (50/30 μm, 2 cm, Sigma-Aldrich, Bellefonte, PA, USA). 

Aroma-active compounds were analyzed by an Agilent 7000 Triple Quadrupole GC-MS system (Santa Clara, CA, USA) equipped with an olfactory detector port (ODP4, Gerstel GmbH, Mühlheim, Germany) and a polar DB-Wax capillary column (30 m × 0.25 mm i.d. × 0.25 μm d_f_, Agilent Technologies, Santa Clara, CA, USA) under a splitless mode [16,17]. The injection temperature was set to 250 °C and the temperature for the olfactometric transfer line was 220 °C to ensure that volatile compounds were active. Pure helium (>99.99%) was used as carrier gas at a flow rate of 2.1 mL/min. The column temperature was programmed as reported by Gu et al. [18] with no modifications. The ion source temperature was held at 240 °C and operated in electron impact mode at 70 eV with a scan range from 41 to 550 *m*/*z*. An effluent split was controlled by a four-way pipe and the injection ratio between the MS detector and the olfactory port was set to 1:1 (*v*/*v*). To avoid nasal mucosa dehydration of panelists during olfactory trials, humidified air was released to the sniffing port at 20 mL/min. 

During evaluation, the panelists were asked to sniff through the olfactory mask. When an aroma-active compound was sniffed and recognized, the panelists were asked to record the perceived aroma characteristics and press the remote-controlled button to record the odor intensities. The intensity was categorized into five grades from 0–4, with “0” indicating no odor, “1”, a weak odor; “2”, a moderate odor; “3”, a strong odor; and “4”, a very strong odor.

Aroma compounds were identified by comparing the retention index (RI), MS spectra, and aroma description recorded in the literature and database [19]. Specifically, the MS data were analyzed with Agilent MassHunter Qualitative Analysis B7.0 software (Agilent, Santa Clara, CA, USA) by comparing the Mass Spectral Library. A series of *n*-alkanes were analyzed under the same chromatographic conditions to calculate RI, which was compared with the records from the NIST Chemistry WebBook (http://webbook.nist.gov/chemistry/, assessed on 14 June 2024. The aroma description was matched to the Flavornet website (www.flavornet.org, assessed on 15 June 2024) and related literature.

### 2.7. Sensory Evaluation

Sensory evaluation was performed according to our previous studies with modifications [15]. A total of 20 healthy panelists (16 females and 4 males, 19–26 years of age) were recruited from the students and staff at the School of Food Science and Technology, Nanjing Agricultural University (Nanjing, Jiangsu, China), following the instructions recorded in ISO 8586:2023 [20] and ISO 5496:2006 [21]. The sensory panel had previous experiences in sensory evaluation and received extra training sessions for evaluating sponge cakes. 

Prior to the evaluation, slices of uniform size were taken from different cake recipes, randomly coded with three-digit numbers, and served on white plastic plates. Spicy food and strong-scented personal care products were also forbidden on the day. The panelists were not allowed to smoke or eat for at least 2 h before the test. Sensory evaluation was performed in a food-grade laboratory at room temperature (25 ± 2 °C). The panelists were asked to rinse their palate with water between different samples to avoid residual effects.

To evaluate if typical pandan attributes were well accommodated in pandan-flavored sponge cakes, the panelists were asked to rate the intensities of three principal attributes, i.e., color (greenness), olfactory properties (fat-like, green/grass-like, tea-like, burnt, and baking aromas), and two gustatory properties (bitterness and sweetness) using a scale from 1.0 (not perceivable) to 10 (strong). In this section, the pandan-flavored cakes were prepared without adding sugar and salt, and the other ingredients were the same as presented in Table 1. 

### 2.8. Statistical Analysis 

Experiments were performed in triplication and values were expressed as mean ± standard deviation. Data were evaluated by one-way analysis of variance (ANOVA) using SPSS Statistics software version 27 (IBM Corporation, Armonk, NY, USA), and significant differences were defined as *p* < 0.05 by Duncan’s multiple-range tests. Graphical representations were generated by Origin 2022 software (Origin Lab Corporation, Northampton, MA, USA).

## 3. Results and Discussion

### 3.1. Effects of Thermal Processing on Typical Pandan Aroma

#### 3.1.1. Comparison of Aroma-Active Compounds in Fresh and Thermal-Treated Pandan Leaf Juice

A total of 20 volatile compounds were detected as odorants from the control (fresh pandan leaf juice) and TPJ-130 (thermal-treated pandan leaf juice samples at 130 °C) samples; among which 15 were tentatively identified by comparing mass spectra and RI with those recorded in the Mass Spectral Library, as well as odor description matching with the NIST webbook, Flavornet website (www.flavornet.org, assessed on 15 June 2024), and the related literature (Table 2). 

The control sample contained 9 odor-active compounds, while after thermal treatment, a total of 17 aromas were detected by the panelists. This is understandable since thermal processing technologies such as baking and drying would result in a higher degree of reaction among aroma precursors. Conspicuously, the general aroma profile of TPJ-130 was distinct from the control sample. Specifically, only 6 compounds were shared in the control and TPJ-130 samples, whereas 11 odorants were only distributed in pandan leaf juices after thermal processing. The commonly present compounds include leaf alcohol (no. 1, green/grassy note), 2AP (no. 2, popcorn-/steamed rice-like note), benzeneacetaldehyde (no. 3, green/grassy note), 1-ethyl-2-pyrrolecarboxaldehyde (no. 4, roasted/steamed rice-like note), *β*-homocyclocitral (no. 5, tea-like/camphoreous/mint-like note), and (*R*)-styralyl alcohol (no. 6, matcha-like/floral note). To note, 2AP was been identified as a key aroma compound in pandan leaves imparting a desirable “popcorn-like” aroma for non-Orientals or “pandan-like” aroma for Orientals [22]. Apart from its occurrence in pandan leaves, the 2AP has been found in many other plants or foodstuffs, such as winter melon, toast, maize flour, roasted sesame, and bread crust [4,23]. Our study revealed that 2AP was present in the fresh and thermal processed samples with similar odor intensity (Table 2). This finding was supported by previous reports that the 2AP was only produced once the pandan leaves were pounded, withered, or cooked [24]. Moreover, compared with the control, the TPJ-130 sample contained higher odor intensities of leaf alcohol and 1-ethyl-2-pyrrolecarboxaldehyde; in contrast, the odor strength of the camphoreous and tea-like notes given by *β*-homocyclocitral was slightly decreased in the TPJ-130. 

There were 11 aroma-active compounds exclusively present in the TPJ-130 sample; three of them cannot be identified due to the incomplete records in the database. Among the identified odorants, 2-penylfuran (no. 12, green bean-/vegetable-like/grassy note), (*E*)-2-decenal (no. 14, green/cucumber-like note), and 3-phenylfuran (no. 17, milky/caramel-like note) were the most powerful aroma candidates in the TPJ-130 sample as their odor intensity values were the highest. There were four odor-active compounds also identified with moderate strength, namely, 2-ethyl-1*H*-pyrrole, dimethyl benzyl carbinol, 1-methoxy-docane, and (*E*)-beta-damascenone. These compounds would enrich the flavor profile of the sample by providing woody, herbaceous, matcha-like, and fruity notes. 2-Ethyl-1*H*-pyrrole was also detected from the supercritical carbon dioxide extract of pandan leaves [22]. Thus far, a whole picture and variations of the aroma profile between fresh and thermal-treated pandan leaf juices was uncovered to a certain degree. 

#### 3.1.2. E-Nose Analysis of Volatile Compounds in Thermal-Treated Pandan Leaf Juices

An *E*-nose apparatus was used to distinguish the control and four pandan leaf juice samples (thermal-treated under 80 °C, 100 °C, 120 °C, and 140 °C, respectively). Slight changes in the composition of volatile compounds induced by different degrees of thermal treatments may lead to a significantly varied sensor response. The *E*-nose technique, therefore, has been applied in various analyzing scenarios from foodstuffs, such as tea [25], strawberry juice [26], and tangerine peel [27].

**Figure 1:**
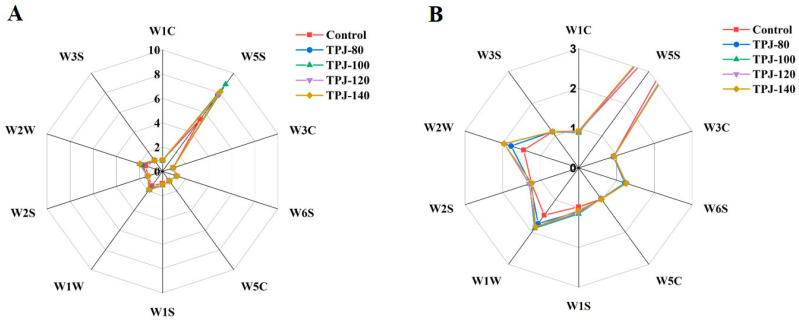
The radar plot of the *E*-nose analyses for fresh and thermal-treated pandan leaf juices under various temperatures. (**A**) Regular scale; (**B**) zoomed-in coordinates. “Control” represents the fresh pandan leaf juice without treatment; “TPJ-80”, “TPJ-100”, “TPJ-120” and “TPJ-140” denote thermal-treated pandan leaf juice under 80 °C, 100 °C, 120 °C, and 140 °C, respectively. W1C, sensitive to aromatics and benzene; W5S, high sensitivity, especially nitrogen oxides; W3C, sensitive to ammonia and aromatic components; W6S, mainly selective for hydrogen; W5C, sensitive to alkane aromatic components; W1S, sensitive to short-chain alkanes and methane; W1W, sensitive to inorganic sulfides; W2S, Sensitive to alcohol, ether, aldehydes and ketones; W2W, sensitive to aromatic components and organic sulfides; and W3S, sensitive to long-chain alkanes.

In the current study, the *E*-nose equipped with 10 sensors was applied to analyze the integrated flavor profile of the fresh and thermal-treated pandan leaf juices, and to demonstrate the sensor response data, a radar fingerprint of volatile components was organized in Figure 1. To note, the sensor intensity was not only dependent on the composition of the odor molecules but also on their concentrations. In Figure 1A, the highest response was observed for the W5S sensors, regardless of the thermal-treated temperatures. This indicated that the volatile compounds of fresh and heated pandan leaf juices were mainly nitroxides. As reported by Wakte et al. [28], 2AP was the major volatile compound for all pandan leave samples collected from the eastern and western coastal regions of India, accounting for over 12% of the total volatile composition. Other nitroxides such as *n*-methylmethacrylamide, pyrrolidine, and 4*H*-Pyran-4-one were also reported to be present in the ultrasonic extract of pandan leaves using ethanol as a solvent [22]. In comparison, the sensors of W1C, W3C, and W5C showed weak signal intensities, suggesting low levels of volatile compounds in the categories of aliphatic aromatics, ammonia, alkanes and aromatic components. A zoomed-in figure was further constructed in Figure 1B to demonstrate the sensor responses to five samples. The signal intensities of different sensors for the same pandan leaf juice sample were significantly different, and the sensor signal intensity of the control and thermal-heated samples by the same sensor also presented differences, especially for W2W, W1W, and W1S. Previous studies reported that W2W was sensitive to sulfuric organic compounds; W1W was sensitive to many terpenes and sulfur organic compounds (i.e., limonene, pyrazine); W1S was sensitive to methane compounds [15]. Furthermore, the radar fingerprint chart of the four thermal-treated pandan leaf juices under 80 °C, 100 °C, 120 °C, and 140 °C were completely overlapped for the W1C, W3C, W5C, and W6S sensors, suggesting minimal temperature impacts on the levels of aromatic, ammonia, alkanes, and hydrogen compounds. Though *E*-nose is a fast technique that quickly assign volatile compounds to certain categories, it cannot identify specific components or provide quantitative data for comparison. Therefore, to fully resolve the differences in aroma and flavor attributes among samples, state-of-the-art technologies such as GC-IMS should be utilized to provide digital fingerprints through pattern recognition.

#### 3.1.3. GC-IMS Analysis of Volatiles in Pandan Leaf Juices upon Different Degrees of Thermal Treatments

**Figure 2:**
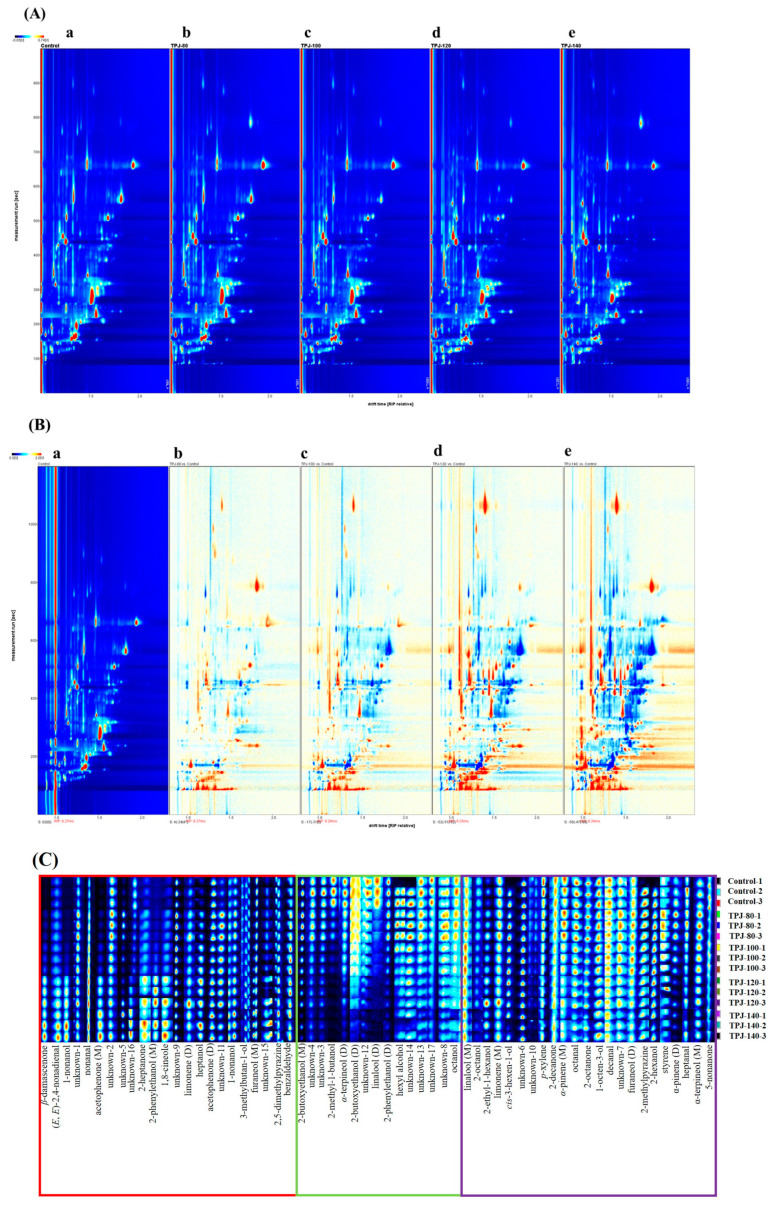
HS-GC-IMS analysis of fresh and thermal-treated pandan leaf juices. (**A**) Topographic plot of flavor compounds; Group a: control (fresh pandan leaf juice); Group b: “TPJ-80”, thermal-treated pandan leaf juice under 80 °C; Group c: “TPJ-100”, thermal-treated pandan leaf juice under 100 °C; Group d: “TPJ-120”, thermal-treated pandan leaf juice under 120 °C; Group e: “TPJ-140”, thermal-treated pandan leaf juice under 140 °C; (**B**) difference comparison plot using the spectrum of control (fresh pandan leaf juice) as the reference; (**C**) fingerprints spectra of fresh and the four thermal-treated pandan leaf juice samples generated by Gallery Plot.

A direct comparison of the spectral differences for the fresh and thermal-treated pandan leaf juices under different temperatures was achieved using the reporter plugin software (Figure 2). Figure 2A presents a two-dimensional GC-IMS topographic map, in which the abscissa and ordinate represent the ion drift time and retention time in gas chromatography, respectively. In the figure, each spot denotes a volatile compound and the color depth is directly proportional to its content, for example, the red color represents a high concentration whereas the white color represents a low compound level. As indicated in Figure 2A, the GC-IMS technique efficiently separated the signal peaks from the samples, the retention time was primarily between 100–700 s, and the drift time was 1.0 to 2.0 ms. Based on the GC-IMS topographic plot in Figure 2A, it can be found that the peak number of volatile compounds in the control and four samples were generally similar but their peak intensity (i.e., the volatile content) was slightly varied. To compare this difference between the samples, discrepancy images were constructed as presented in Figure 2B, in which the spectrum of the control (a) was adopted as a reference and the sample spectrum (b–e) was subtracted from this reference. The subtracted background appeared white if the flavor components were consistent; the red color indicated that the compound was present at a higher concentration in the analyzed sample, whereas blue signals denoted a lower value. Figure 2B showed that when using fresh pandan leaf juice as a reference, there were significant differences in the composition and amount of the flavor compounds in the thermal-treated samples under various temperatures, i.e., 80 °C, 100 °C, 120 °C, and 140 °C. Particularly, in TPJ-120 and TPJ-140 (fresh pandan juices heated under 120 °C and 140 °C, respectively), most substances showed significantly increased concentrations. As reported by Wang et al., thermal processing treatment may result in denaturation of macromolecules, acerating possible interactions of volatile compounds with proteins, polyphenols, and/or carbohydrates in the juice sample [29]. This might explain the appearance of darker blue spots in the Figure 2B. 

The flavor fingerprint spectra for the fresh and thermal-treated samples were generated using the Gallery Plot plugin (FlavourSpec^®^ GC-IMS instrument, Dortmund, Germany), and the results are presented in Figure 2C. A total of 45 types of volatile compounds were identified based on their retention indices and drift times recorded in the GC-IMS and NIST libraries. Due to incomplete databases, it is impossible to characterize all compounds detected by GC-IMS. Consequently, 15 substances were listed as unidentified compounds in the Supplementary Materials together with their formula and molar mass. 

In Figure 2C, each column denotes the same flavor compounds across different pandan samples, whereas each row represents the sample analyzed. The red-framed area of Figure 2C shows an abundance of volatile compounds in the thermal-processed pandan juices under relatively high temperatures (TPJ-120 and TPJ-130), including *β*-damascenone, (*E*,*E*)-2,4-nonadienal, 1-nonanol, 2-heptanone, heptanol, and 1,8-cineole, etc., as well as several unidentified compounds, i.e., unknown-11 (C_10_H_18_O, M_w_ 154.3), unknown-5 (C_10_H_23_NS, Mw 189.4), and unknown-15 (C_4_H_10_O_2_S, M_w_ 122.2) (Supplementary Table S1). Notably, these compounds were almost absent in the fresh samples but gradually appeared in the heat-treated juices as the temperature increased. The presence of alcohols and ketones might be related to slower aldehyde oxidations or alcohol degradation upon thermal treatments. Figure 2C suggests that some substances such as acetophenone (D), heptanol, furaneol (M), and benzaldehyde gradually accumulated with an increase in heating temperature. Among them, benzaldehyde was widely distributed in many foods, for example, tamarillo juice [16], fermented edible fungus beverage [15], barley malts [18], and Jiuqu hongmei tea [24], contributing to a bitter almond odor. In the area labeled with a green rectangle, some typical volatile compounds including *α*-terpineol (D), 2-phenylethanol (D), hexyl alcohol, linalool (D), and octanol were predominant in the control sample but gradually decreased in concentration with thermal processing. Previous studies have indicated that *α*-terpineol, linalool, and 2-phenylethanol contribute to the characteristic woody, pleasant citrus-, and rose-like odors; however, these compounds were not identified as odor-active compounds during GC-O-MS analyses in Section 3.1.1, which might be ascribed to their lower concentrations as compared to the odor thresholds. The purple-framed area of Figure 2C reveals that the levels of several volatile substances changed slightly in the control and heated pandan leaf juice samples, regardless of processing temperatures. These included linalool (M), 2-ethyl-1-hexanol, limonene (M), *cis*-3-hexen-1-ol, *p*-xylene, 2-decanone, α-pinene (M), octanal, 2-octanone, 1-octen-3-ol, decanal, methylpyrazine, 2-hexenol, styrene, and 5-nonanone, etc., as well as some unidentified substances, i.e., unknown ***6***, ***10***, ***7*** (Supplementary Table S1). 

Detailed results of identified compounds with their peak volumes as obtained from the GC-IMS analyses of five samples are summarized in Table 3. These compounds were further categorized into six groups, including 10 ketones, 16 alcohols, 6 aldehydes, 10 terpene-derivates, 2 pyrazines, and 1 other compound. The volatile compounds listed in Table 3 were markedly different than the odor-active compounds as summarized in Table 2. The possible explanations were as follows: (1) some analytes were excluded in Table 2 since they were only detected and/or identified by MS but not the sniffing port, which means they were not odor-active compounds; (2) many substances detected by GC-IMS cannot be identified due to database limitations; and (3) GC-IMS techniques showed advantages of no pre-treatments and low detection limits, indicating their abilities to detect more types of volatile molecules.

During GC-IMS analyses, volatile compounds were evaporated from the heated sample and then transferred into the ionization zone of the IMS drift tube via carrier gas, in which various ions were generated with different drift times [30,31]. In the ionization region, certain compounds may produce multiple signals, forming monomers, dimers, or even trimers, and this was mainly dependent on the concentrations of volatile substances and their half-life in the drift tube [30,32]. In the current study, there were eight volatile compounds containing monomers and dimers, which were acetophenone (***3*** and ***4***), furaneol (***8*** and ***9***), 2-phenylethanol (***18*** and ***19***), and 2-butoxyethanol (***24*** and ***25***), *α*-pinene (***33*** and ***34***), limonene (***37*** and ***38***), *α*-terpineol (***39*** and ***40***), and linalool (***41*** and ***42***). 

As presented in Table 3, the percentage of total ketones was significantly increased from 15.23% (percentage calculated using the total peak volume of all identified volatile substances) in the fresh juice to 23.41% in TPJ-140, which was mainly ascribed to the substances of *β*-damascenone (***1***, peak volume: 256.53 to 2615.21), acetophenone (M) (***3***, peak volume: 45.58 to 656.26), and 2-heptanone (***7***, peak volume: 194.03 to 637.7). Among them, *β*-damascenone is an important fragrance chemical widely used in the perfume industry, contributing to a pleasant rose-like aroma despite being present in low concentrations. As mentioned by Yang et al. [33], heat processing together with acidity and light treatments enhanced the formation of damascenone in tea. On the other hand, terpene-derivates were the major volatiles in fresh and heated pandan samples regardless of the thermal-processing temperatures, accounting for over 24% of the total identified volatile substances. The fresh sample presented the highest amount of total terpene-derivates (39.07%) and after heating, this value dropped to 24.22% in TPJ-140. In this group, *α*-terpineol (***38***), with woody and floral scents decreased significantly, especially in the dimer form, from a peak volume of 8512.63 (control) to 1231.98 (TPJ-140). Similarly, the dimer form of linalool constantly decreased in concentration with increased heating temperature (80 °C to 140 °C). Overall, Table 3 clearly shows the changes of identified volatile compounds across fresh and four thermal-treated samples; however, if pandan-derived flavor compounds can be well accommodated in the real foodstuff of sponge cake, and whether these changes would impact the overall sensory attributes of pandan-flavored products is still an open question that needs to be further explored. 

### 3.2. Effect of Pandan Leaf Juice on the Physical Properties of Batter and Cake 

Batter density is associated with the ability of the ingredient mixture to capture and hold air during the mixing process. The batter density of pandan-flavored sponge cake supplemented with different levels of pandan leaf juices was shown in Supplementary Figure S1. Overall, the batter density gradually decreased with the increasing additions of pandan juice, from 0.641 in PFC-1 to 0.384 in PFC-4 (*p* < 0.05). This follows the findings of Díaz-Ramírez [34] who partially replaced the egg white protein with whey protein isolate during sponge cake preparation and those of Paraskevopoulou et al. [35] who used a maize-milling by-product for wheat flour substitution. This phenomenon might be related to the increased amount of moisture in the cake batter supplemented with higher levels of pandan leaf juice, which is beneficial for the foaming capacities of eggs. The reduction in batter density is associated with the higher final volume and a soft texture of the final sponge cake. 

All color data were expressed as L* (brightness), a* (greenness to redness), and b* (blueness to yellowness) values, and △E was calculated using the sponge cake without pandan addition as a reference. From Table 4, both the crust and crumb color were affected by the addition of pandan leaf juice to the sponge cake recipe. In general, the progressive increase in the level of pandan leaf juice resulted in an decreased brightness (L*) of the cake crust and crumb compared to the control group (Table 4). Similar findings were reported by Lu et al. [36], in which sponge cake samples with 10%, 20%, and 30% green tea powder substitution were darker than the control (0% green tea powder addition). The crust color of the cake was mainly related to the Maillard reaction of sucrose and protein and/or sugar caramelization [37]; during the baking/heating process, the moisture content on the surface drops sharply, which provides the best conditions for these reactions, thereby generating typical compounds with intense brown color. Furthermore, the crust remained in the red space, though it significantly decreased from 12.02 in control to a value of 7.35 in the PFC-4 sample. It is worth noting that the attractive green color of the pandan leaves was only observed in the cake crumb (Table 4), in which the value of a* decreased from 6.27 to −9.61 in the pandan-flavored sponge cake. Similarly, the yellowness (reflected by b*) of the crumb of the four pandan-flavored cake samples was significantly higher than that of the control (42.49 ± 1.58), especially for the PFC-2 and PFC-3 samples (48.13 ± 0.58 and 48.42 ± 0.73, respectively); while no significant differences of b* were observed for the cake crust, excepted for the sample supplemented with 10.3% of pandan leaf juice (PFC-1, 36.36 ± 1.07). Overall, the results showed that increasing the level of pandan leaf juice makes the cake crumb darker and greener. 

**Figure 3:**
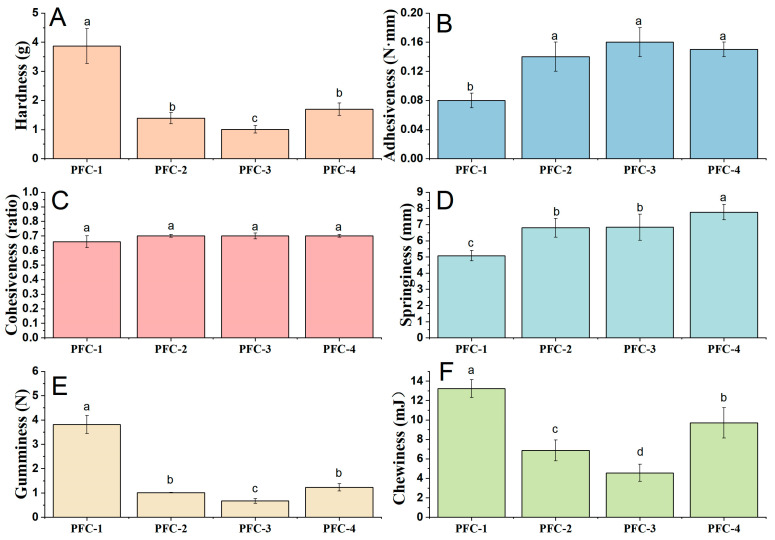
TPA parameters of pandan-flavored sponge cake with varied formulations. (**A**) Hardness; (**B**) adhesiveness; (**C**) cohesiveness; (**D**) springiness; (**E**) gumminess; (**F**) chewiness. PFC-1, PFC-2, PFC-3, and PFC-4: pandan-flavored sponge cake samples supplemented with 10.3%, 13.3%, 16.1%, and 18.8% pandan leaf juice, respectively. Different letters within the figure indicate significant statistical differences using Duncan’s multiple range test by one-way ANOVA analysis (*p* < 0.05).

For texture profile analyses, the hardness values indicated that the sponge cake became softer with the increasing levels of pandan leaf juices (Figure 3A). The hardness of the cake is directly related to the density of the tested materials. The weight of each cake sample in this study was slightly different (as shown in Table 1), which may be the reason for the decrease in hardness. For cohesiveness, there were no significant differences (*p* > 0.05) among four pandan-flavored cake samples, which suggested that the cake sample retained a similar form between the first and second chew. The PFC-2, PFC-3, and PFC-4 showed significantly higher values for adhesiveness than PFC-1. Springiness is an important textural parameter related to the elasticity of the sample. The parameter was measured by determining the degree of recovery between the first and second compression (stimulating the first and second bite during oral processing). TPA results showed an increase in cake springiness with an increased level of pandan leaf juice (Figure 3D), and the highest value was observed for the PFC-4 sample, which might require more mastication energy in the mouth. Gumminess is defined as the product of cohesiveness and hardness, while chewiness (a measure of the energy required to masticate food for swallowing) is calculated by multiplying gumminess by springiness. These two parameters showed higher values in pandan-flavored cakes than the control, especially in the PFC-3 cake which was characterized by significantly lower values of gumminess and chewiness. Overall, the TPA results demonstrated an increase in cake adhesiveness and springiness and a decrease in hardness, gumminess, and chewiness. 

### 3.3. The Role of Thermal Processing on Typical Pandan-Featured Aroma in Sponge Cake 

#### 3.3.1. Sensory Evaluation of Pandan-Flavored Cakes Prepared under Various Temperatures

**Figure 4:**
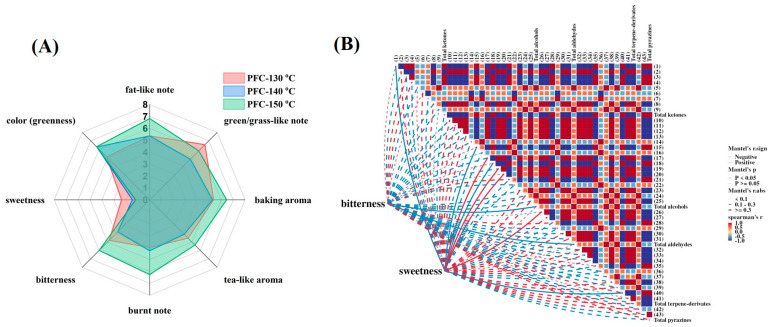
(**A**) Sensory evaluation results of typical pandan-related features in sponge cakes; (**B**) correlation heatmap analysis representing the possible correlations between volatile compounds and important taste attributes (bitterness and sweetness) of pandan-flavored sponge cake.

To comprehensively evaluate if the baking temperatures would influence the fitness and the retention of typical pandan attributes in sponge cakes, the cake samples were prepared under different baking temperatures, 130 °C, 140 °C, and 150 °C. Since the additional sugar and salt would hinder the accurate evaluation of pandan-related features, the pandan-flavored sponge cakes (containing 18.8% of pandan leaf juice) used for sensory evaluation were enhanced without adding sugar and salt. Three major pandan-related characteristics, including color (green-ness), olfactory properties (fat-like, green/grass-like, tea-like, burnt, and baking aromas), and two gustatory properties (bitterness and sweetness) were evaluated. Results were constructed in a radar plot in Figure 4A. Generally, the greenness ratings were consistent with the values measured by chromameter (data was shown in Supplementary Table S2). Specifically, the pandan-flavored sponge cake prepared under 130 °C was greener than the PFC-140 °C and PFC-150 °C samples both during sensory evaluation and color measurement (PFC-130 °C vs. PFC-140 °C vs. PFC-150 °C, ratings: 5.1 vs. 6.27 vs. 6.30, a*: −7.56 vs. −8.83 vs. −10.12). Compared with the mild thermal processing conditions (130 °C and 140 °C), the sponge cake baked under a relatively higher temperature (150 °C) showed stronger fat-like, baking, tea-like, and an unpleasant burnt note as well as a prominent bitter taste. As for the sweetness, a progressive decrease was observed in the perceived cake sweetness when the baking temperature increased from 130 °C to 140 °C. Compared to the sweetness, the pandan-flavored sponge cakes showed a distinct bitter taste and the rating scores ranged from 3.8 (PFC-130 °C) to 6.0 (PFC-150 °C). The bitter taste might be related to the presence of bitter and/or astringent substances in pandan leaves, including, but not limited to, phenolics/polyphenols, amino acids, organic acids, terpenoids and alkaloids [38]. Future work is required to identify bitter components from pandan leaves using sensory-guided approaches such as molecular sensory science, taste dilution analysis, dose-over-threshold factors, etc. 

#### 3.3.2. The Possible Contributions of Pandan Volatile Compounds to the Sweet and Bitter Tastes of Sponge Cake

The sense of smell has been reported to contribute more to flavor perception than taste, as more than 80% of food taste is derived from the nose [39,40]. Volatile compounds not only determine the odor profile of a given food but also interact with other senses such as taste and texture to further modulate the ultimate flavor perception [41]. Therefore, in this section, the possible contributions of pandan volatile compounds to the sweetness and bitterness of sponge cake were evaluated by a correlation heatmap using the peak volume of volatile compounds listed in Table 3. 

As shown in Figure 4B, four volatile compounds might be positively correlated with the sweet taste, including 2-phenylethanol (***17***), 2-methyl-1-butanol (***19***), hexyl alcohol (***20***), and decanal (***26***). Similarly, the levels of total alcohol and total aldehydes were suggested to be correlated with the perceived bitterness of the pandan-flavored sponge cake (Figure 4B). Sweetness enhancement by aromas is a promising strategy to mitigate sugar reduction in food products, and many odorants have been found to exhibit the ability to enhance the perceived strength of sweetness. For example, Liu et al. [42] reported that 2-furanmethanol, 2-methoxyphenol, 2,3-butanedione, benzeneacetaldehyde, and furfural dramatically enhanced the intensity of a sugar solution. Xiao et al. [43] also observed the odor-induced sweetness enhancement phenomenon for the volatile compounds of 2-methylbutyl acetate, ethyl acetate, 3-methylbutyl butanoate, and hexyl acetate in 30 g/L sucrose solution. Similarly, some volatile compounds in foodstuffs are also able to impact the perception of bitter taste, such as the decreased bitterness of wine by volatile fruity compounds [44], low bitterness by the addition of hop aroma [45], and the increase in bitter taste of olive oil by the cut-grass odorant of *cis*-3-hexen-1-ol [46]. To note, this study only predicted the possible relationships of volatile compounds of pandan leaves and the taste traits of pandan-flavored sponge cake by statical analyses of correlation heatmaps. The odor-induced taste enhancement phenomena should be further verified by sensory-guided approaches in the model solution and real food matrices. 

## 4. Conclusions

The impacts of thermal treatment on the aroma profiles of pandan leaf juices were comprehensively analyzed using GC-O-MS, *E*-nose, and GC-IMS analyses. Generally, the heating process enriched the odor-active compounds in pandan leaf juice by enhancing the odor intensities of the original volatiles (especially for the roasted and steamed rice-like odors) or introducing more types of odorants with pleasant aroma characteristics (a total of 9 odor-active compounds for fresh juice and 17 for the heated juice sample). The peak volume as analyzed by GC-IMS indicated that thermal processing under different temperatures had a positive impact on the aroma profiles and facilitated the formation of ketones, alcohols, and aldehydes. The addition of pandan leaf juice resulted in a decreased brightness (L*) of the cake crust; the attractive green color of pandan leaves was only apparent in the cake crumb according to the a* spaces and the photo records. For TPA parameters, the sponge cake supplemented with 16.1% pandan leaf juice demonstrated advantages in hardness, gumminess, and chewiness properties. As revealed by the correlation heatmap, several volatile compounds (i.e., 2-phenylethanol, 2-methyl-1-butanol, hexyl alcohol, decanal) might be keys to modulating the flavor attributes of pandan-flavored sponge cake as they were highly correlated with sweetness; however, their odor-induced taste enhancement abilities required more validation.

## Figures and Tables

**Table 1:** Formulation of the sponge cakes.

Ingredient	Manufacture	Control ^a^	PFC-1	PFC-2	PFC-3	PFC-4
pandan leaf juice	—	0	8.4	11.2	14	16.8
whole egg (g) (egg yolk and egg white were then separated for usage)	Hangzhou Qiandao Lake Zhikang Agricultural Development Co., Ltd., Hangzhou, China	43	43	43	43	43
corn starch (g)	Yantai Shuangta Foods Co., Ltd., Yantai, China	2.2	2.2	2.2	2.2	2.2
low gluten flour (g)	Shandong Fushikang Noodle Group Co., Ltd., Feicheng, China	12	12	12	12	12
corn oil (g)	Golden Sun Cereals and Oils Co., Ltd., Nantong, China	3.3	3.3	3.3	3.3	3.3
butter (g)	Fonterra Trading Co., Ltd., Shanghai, China	3.3	3.3	3.3	3.3	3.3
sodium chloride (g)	Jiangsu Salt Industry Group Co., Ltd., Nanjing, China	0.1	0.1	0.1	0.1	0.1
sugar	Shanghai Delfu Sugar Co., Ltd., Shanghai, China	9	9	9	9	9

^a^ Control, PFC-1, PFC-2, PFC-3, and PFC-4: Pandan-flavored sponge cake samples supplemented with 0%, 10.3%, 13.3%, 16.1%, and 18.8% pandan leaf juice, respectively.

**Table 2:** Identification of odor-active compounds in fresh and thermal-treated pandan leaf juice samples using GC-O-MS analyses.

No.	CAS	Compound	Odor Description	Odor Intensity	
				Control	TPJ-130 ^a^
1	928-96-1	leaf alcohol	green, grassy	3	4
2	85213-22-5	2AP	popcorn-, steamed rice-like	4	4
3	122-78-1	benzeneacetaldehyde	green, grassy	4	4
4	2167-14-8	1-ethyl-2-pyrrolecarboxaldehyde	roasted, steamed rice-like	2	4
5	472-66-2	*β*-homocyclocitral	tea-like, camphoreous, mint-like	4	3
6	1517-69-7	(*R*)-styralyl alcohol	matcha-like, floral	3	3
7	1568-20-3	5-methyl-2-pyrazoline	milky	2	—
8	—	unknown-1	sweet	3	—
9	—	unknown-2	bitter melon	3	—
10	763-32-6	3-methyl-3-buten-1-ol	burnt	—	2
11	1551-06-0	2-ethyl-1*H*-pyrrole	woody	—	3
12	3777-69-3	2-pentylfuran	green bean-, vegetable-like, grassy	—	4
13	100-86-7	dimethyl benzyl carbinol	bitter, herbaceous, mild floral	—	3
14	3913-81-3	(*E*)-2-decenal	green, cucumber-like	—	4
15	—	unknown-3	raw chili pepper-like	—	4
16	7289-52-3	1-methoxy-docane	fresh, matcha-like	—	3
17	13679-41-9	3-phenylfuran	milky, caramel-like	—	4
18	—	unknown-4	raw rice-like	—	2
19	23726-93-4	(*E*)-beta-damascenone	fruity, sweet, honey-like	—	3
20	—	unknown-5	floral, sweet	—	4

^a^ TPJ-130 denotes thermal-treated pandan leaf juice under 130 °C.

**Table 3:** The peak volume of GC-IMS-identified volatile compounds from fresh (control) and thermal-treated pandan leaf juices under various temperatures.

Compounds	GC-IMS Peak Volume				
	Control ^a^	TPJ-80	TPJ-100	TPJ-120	TPJ-140
*Ketones*					
(*1*) *β*-damascenone	256.53 ± 31.36 ^c^	532.54 ± 25.13 ^bc^	827.06 ± 33.02 ^b^	2299.5 ± 148.12 ^a^	2615.21 ± 509.57 ^a^
(*2*) 2-decanone	683.21 ± 20.4 ^a^	633.14 ± 16.54 ^b^	612.8 ± 16.07 ^b^	604.77 ± 12.72 ^bc^	577.77 ± 22.44 ^c^
(*3*) acetophenone (M)	45.58 ± 2.5 ^d^	89.97 ± 7.2 ^d^	147.44 ± 8.68 ^c^	452.46 ± 15.45 ^b^	656.26 ± 59.75 ^a^
(*4*) acetophenone (D)	1668.46 ± 23.6 ^c^	1865.68 ± 45.56 ^a^	1496.95 ± 18.05 ^d^	1773.98 ± 50.37 ^b^	1930.2 ± 81.02 ^a^
(*5*) 2-octanone	486.61 ± 18.17 ^a^	483.59 ± 12.34 ^a^	413.88 ± 9.16 ^a^	421.12 ± 29.76 ^a^	407.69 ± 95.38 ^a^
(*6*) 5-nonanone	318.91 ± 25.73 ^a^	228.7 ± 3.02 ^b^	306.11 ± 5.45 ^a^	230.68 ± 11.8 ^b^	256.91 ± 42.02 ^b^
(*7*) 2-heptanone	194.03 ± 26.49 ^c^	333.42 ± 20.01 ^b^	332.98 ± 10.12 ^b^	642.89 ± 31.47 ^a^	637.73 ± 118.41 ^a^
(*8*) furaneol (M)	276.26 ± 2.65 ^b^	303.1 ± 10.37 ^a^	299.15 ± 5.26 ^a^	264.44 ± 6.75 ^b^	248.01 ± 9.11 ^c^
(*9*) furaneol (D)	3570.36 ± 187.71 ^a^	3787.35 ± 72.6 ^a^	3620.59 ± 26.26 ^a^	4072.94 ± 110.59 ^a^	3571.72 ± 769.08 ^a^
*Total ketones*	*7499.95 ± 10.02 ^e^*	*8257.49 ± 10.02 ^c^*	*8056.96 ± 10.02 ^d^*	*10,762.79 ± 10.02 ^b^*	*10,901.49 ± 10.02 ^a^*
*Alcohols*					
(*10*) octanol	95.99 ± 4.95 ^a^	103.96 ± 6.68 ^a^	80.2 ± 3.92 ^b^	71.41 ± 3.14 ^c^	56.05 ± 3.08 ^d^
(*11*) 2-octanol	400.4 ± 25.9 ^a^	389.49 ± 14.97 ^ab^	348.75 ± 15.81 ^bc^	342.47 ± 33.86 ^c^	252.41 ± 26.93 ^d^
(*12*) 2-hexanol	447.6 ± 328.28 ^a^	665.35 ± 53.09 ^a^	670.04 ± 8.8 ^a^	615.11 ± 34.32 ^a^	506.36 ± 128.35 ^a^
(*13*) *cis*-3-hexen-1-ol	150.83 ± 100.74 ^a^	231.51 ± 34.36 ^a^	248.33 ± 18.09 ^a^	220.88 ± 30.07 ^a^	161.56 ± 58.76 ^a^
(*14*) 2-ethyl-1-hexanol	423.18 ± 76.09 ^b^	795.94 ± 135.39 ^ab^	709.47 ± 55.39 ^ab^	1005.74 ± 412 ^a^	780.86 ± 236.49 ^ab^
(*15*) 2-phenylethanol (M)	91.98 ± 3.19 ^b^	100.88 ± 13.39 ^b^	209.74 ± 42.4 ^b^	807.47 ± 92.27 ^a^	945.32 ± 217.94 ^a^
(*16*) 1-pentanol	77.64 ± 3.84 ^c^	168.52 ± 8.88 ^bc^	271.66 ± 30.52 ^b^	729.32 ± 82.71 ^a^	620.65 ± 155.35 ^a^
(*17*) 2-phenylethanol (D)	1061.48 ± 83.62 ^a^	963.97 ± 38.83 ^b^	558.75 ± 2.98 ^c^	389.46 ± 17.73 ^d^	318.39 ± 62.3 ^d^
(*18*) 1-nonanol	496.25 ± 34.85 ^bc^	422.97 ± 23.81 ^c^	455.58 ± 40.78 ^c^	605.29 ± 30.93 ^ab^	657.11 ± 117.76 ^a^
(*19*) 2-methyl-1-butanol	537.67 ± 21.17 ^a^	227.76 ± 16.3 ^b^	106.18 ± 6.24 ^c^	67.94 ± 9.55 ^d^	54.44 ± 4.54 ^d^
(*20*) hexyl alcohol	88.02 ± 64.79 ^ab^	132.2 ± 16.23 ^a^	77.4 ± 17.49 ^ab^	61.2 ± 9.78 ^b^	55.96 ± 24.6 ^b^
(*21*) 3-methylbutan-1-ol	694.19 ± 24.44 ^c^	690.84 ± 8.5 ^c^	746.54 ± 8.99 ^c^	909.47 ± 25.66 ^b^	1189.17 ± 127.88 ^a^
(*22*) heptanol	668.4 ± 41.84 ^c^	1235.78 ± 115.11 ^bc^	1893.25 ± 115.64 ^ab^	2469.77 ± 303.29 ^a^	2257.36 ± 878.16 ^a^
(*23*) 2-butoxyethanol (D)	4509.06 ± 605.68 ^b^	5547.11 ± 107.05 ^a^	3423.92 ± 101.07 ^c^	2560.82 ± 195.84 ^d^	1352.17 ± 462.99 ^e^
(*24*) 2-butoxyethanol (M)	1794.25 ± 107.52 ^bc^	2118.78 ± 41.2 ^a^	2045.24 ± 39.64 ^ab^	2053.73 ± 89.41 ^ab^	1674.17 ± 337.83 ^c^
(*25*) 1-octen-3-ol	445.87 ± 21.9 ^a^	429.77 ± 19.8 ^a^	412.8 ± 12.87 ^a^	403.38 ± 28.03 ^a^	394.09 ± 58.78 ^a^
*Total alcohols*	*11,982.82 ± 833.01 ^ab^*	*14,224.85 ± 50.74 ^a^*	*12,257.85 ± 223.69 ^ab^*	*13,313.47 ± 989.98 ^a^*	*11,276.07 ± 2038.28 ^b^*
*Aldehydes*					
(*26*) decanal	848.05 ± 28.49 ^a^	759.64 ± 4.18 ^bc^	806.3 ± 38.78 ^ab^	738.44 ± 19.65 ^c^	717.73 ± 45.3 ^c^
(*27*) benzaldehyde	984.61 ± 7.33 ^b^	1245.57 ± 42.05 ^a^	1435.46 ± 18.84 ^a^	1291 ± 102.74 ^a^	1286.89 ± 270.84 ^a^
(*28*) (*E*,*E*)-2,4-nonadienal	414.42 ± 18.48 ^d^	482.43 ± 5.31 ^cd^	521.52 ± 11.65 ^c^	742.84 ± 74.74 ^b^	872.93 ± 80.16 ^a^
(*29*) nonanal	1781.13 ± 94.72 ^d^	2730.86 ± 97.77 ^c^	3375.85 ± 211.45 ^b^	3861.62 ± 73.73 ^a^	3485.29 ± 226.02 ^b^
(*30*) octanal	1774.66 ± 57.58 ^a^	1809.86 ± 56.02 ^a^	1531.91 ± 6.47 ^b^	1335.12 ± 82.3 ^c^	1042.07 ± 167.92 ^d^
(*31*) heptanal	554.83 ± 139.89 ^a^	567.86 ± 38.52 ^a^	425.46 ± 25.99 ^ab^	392.42 ± 79.66 ^ab^	350.68 ± 163.42 ^b^
*Total aldehydes*	*6357.7 ± 217.36 ^d^*	*7596.23 ± 126.51 ^c^*	*8096.5 ± 164.07 ^ab^*	*8361.43 ± 231.89 ^a^*	*7755.58 ± 291.64 ^bc^*
*Terpene-derivates*					
(*32*) *α*-pinene (M)	281.44 ± 27.31 ^a^	289.95 ± 9.39 ^a^	224.52 ± 6.72 ^b^	187.87 ± 15.02 ^bc^	169.46 ± 36.71 ^c^
(*33*) *α*-pinene (D)	730.91 ± 27.61 ^c^	1084.56 ± 20.74 ^a^	945.84 ± 19.75 ^b^	865.14 ± 38.31 ^b^	712.13 ± 111.62 ^c^
(*34*) styrene	553.31 ± 53.71 ^b^	692.97 ± 40.58 ^a^	563.07 ± 19.57 ^b^	413.21 ± 28.49 ^c^	328.91 ± 114.61 ^c^
(*35*) 1,8-cineole	266.15 ± 47.73 ^b^	479.34 ± 14.16 ^b^	506.04 ± 17.35 ^b^	959.95 ± 223.53 ^a^	1035.79 ± 307.85 ^a^
(*36*) limonene (M)	315.41 ± 62.81 ^b^	519.99 ± 86.43 ^ab^	464.77 ± 41.03 ^ab^	613.76 ± 230.81 ^a^	528.8 ± 107.24 ^ab^
(*37*) limonene (D)	1445.07 ± 53.67 ^a^	1480.19 ± 74.13 ^a^	1154.34 ± 32.53 ^b^	1311.73 ± 265.12 ^ab^	1077.21 ± 76.98 ^b^
(*38*) *α*-terpineol(D)	8512.63 ± 661.4 ^a^	7957.81 ± 142.84 ^a^	3322.49 ± 121.13 ^b^	1868.37 ± 180.87 ^c^	1231.98 ± 226.75 ^d^
(*39*) *α*-terpineol (M)	5380.16 ± 114.13 ^b^	6009.12 ± 45.76 ^a^	5576.21 ± 82.96 ^b^	5607.12 ± 25.56 ^ab^	5297.91 ± 471.92 ^b^
(*40*) linalool (M)	1633.42 ± 32.32 ^d^	2057.24 ± 36.88 ^ab^	1734.18 ± 37.72 ^c^	2024.16 ± 24.57 ^b^	2134.82 ± 70.44 ^a^
(*41*) linalool (D)	129.17 ± 6.08 ^a^	49.26 ± 1.23 ^b^	36.87 ± 2.38 ^c^	20.58 ± 5.23 ^d^	18.5 ± 3.77 ^d^
*Total terpene-derivates*	*19,247.67 ± 1003.87 ^a^*	*20,620.41 ± 224.58 ^a^*	*14,528.33 ± 229.45 ^b^*	*13,871.89 ± 920.58 ^bc^*	*12,535.5 ± 974.71 ^c^*
*Pyrazines*					
(*42*) 2-methylpyrazine	153.65 ± 109.18 ^a^	202.45 ± 5.6 ^a^	193.84 ± 5.74 ^a^	195.21 ± 6.24 ^a^	189.74 ± 18.33 ^a^
(*43*) 2,5-dimethylpyrazine	2877.64 ± 189.37 ^a^	2939.17 ± 89.4 ^a^	2957.26 ± 67.68 ^a^	3068.18 ± 167.17 ^a^	3137.65 ± 170.8 ^a^
*Total pyrazines*	*3031.29 ± 206.13 ^a^*	*3141.62 ± 90.89 ^a^*	*3151.1 ± 71.26 ^a^*	*3263.38 ± 163.47 ^a^*	*3327.39 ± 188.99 ^a^*
*Others*					
(*44*) *p*-xylene	1140.36 ± 97.68 ^a^	1033.57 ± 20.7 ^ab^	908.7 ± 4.35 ^bc^	889.61 ± 33.5 ^bc^	769.88 ± 140.8 ^c^
*Total identified volatiles*	*49,259.78 ± 2368.08 ^ab^*	*54,874.17 ± 523.44 ^a^*	*46,999.43 ± 702.84 ^b^*	*50,462.57 ± 2349.44 ^ab^*	*46,565.92 ± 3644.44 ^b^*

^a^ “Control” represents the fresh pandan leaf juice without treatment; “TPJ-80”, “TPJ-100”, “TPJ-120” and “TPJ-140” denotes thermal-treated pandan leaf juice under 80 °C, 100 °C, 120 °C, and 140 °C, respectively; the same row with different letters denotes significant difference at the level of 0.05.

**Table 4:** Color parameters of sponge cakes prepared with different levels of pandan-leaf juices.

Measurement Site	Sample	L*	a*	b*	△E	Appearance
crust	^a^ Control	66.91 ± 2.73 ^a^	12.02 ± 1.75 ^a^	38.33 ± 0.93 ^a^	—	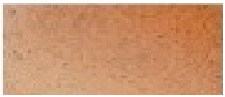
	PFC-1	64.08 ± 1.60 ^a^	9.49 ± 0.54 ^b^	36.36 ± 1.07 ^b^	4.53 ± 0.75 ^d^	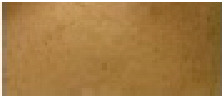
	PFC-2	61.08 ± 0.78 ^b^	8.42 ± 0.91 ^bc^	39.09 ± 0.97 ^a^	7.00 ± 0.29 ^c^	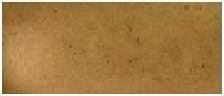
	PFC-3	59.96 ± 0.55 ^b^	7.90 ± 0.55 ^bc^	38.54 ± 0.30 ^a^	8.10 ± 0.22 ^b^	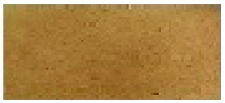
	PFC-4	59.26 ± 1.26 ^b^	7.35 ± 1.04 ^c^	39.08 ± 0.95 ^a^	9.12 ± 0.35 ^a^	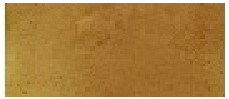
crumb	Control	64.44 ± 0.98 ^a^	6.27 ± 0.63 ^a^	42.49 ± 1.58 ^c^	—	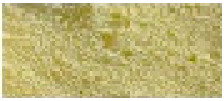
	PFC-1	62.73 ± 1.30 ^ab^	−8.41 ± 0.42 ^b^	45.28 ± 0.63 ^b^	25.54 ± 0.39 ^c^	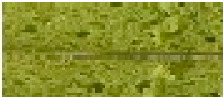
	PFC-2	61.46 ± 1.75 ^b^	−8.65 ± 0.38 ^b^	48.13 ± 0.58 ^a^	28.15 ± 0.57 ^ab^	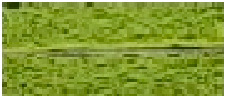
	PFC-3	60.94 ± 0.95 ^b^	−9.06 ± 0.12 ^bc^	48.42 ± 0.73 ^a^	28.65 ± 0.62 ^a^	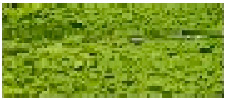
	PFC-4	58.41 ± 0.29 ^c^	−9.61 ± 0.34 ^c^	45.93 ± 0.69 ^b^	27.36 ± 0.64 ^b^	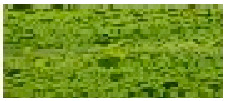

^a^ Control, PFC-1, PFC-2, PFC-3, and PFC-4: pandan-flavored sponge cake samples supplemented with 0%, 10.3%, 13.3%, 16.1%, and 18.8% pandan leaf juice, respectively. In the same row, the different letters denote significant differences at the level of 0.05.

## Data Availability

The original contributions presented in the study are included in the article/Supplementary Materials, further inquiries can be directed to the corresponding author.

## Supplementary Materials

The following supporting information can be downloaded at: https://www.mdpi.com/article/10.3390/foods13193074/s1, Table S1: Unknown substances detected by GC-IMS analyses and their relative content (%); Table S2: Color parameters of sponge cakes prepared with different baking temperatures; Figure S1: The batter density of Pandan-flavored sponge cake samples supplemented with 0%, 10.3%, 13.3%, 16.1% and 18.8% of Pandan leaf juice, respectively.
