# Supplementary material for: Quality-Driven Design of Pandan-Flavored Sponge Cake: Unraveling the Role of Thermal Processing on Typical Pandan Aroma
Source: Foods. 2024 Sep 26;13(19):3074. doi: 10.3390/foods13193074 (PMC11475937; doi:10.3390/foods13193074)
Supplement: Supplementary file 1 [file foods-13-03074-s001.zip › foods-3201107-supplementary.pdf]

Supplementary Table S1 Unknown substances detected by GC-IMS analyses and their relative content (%)

Compound	Formula	Molar mass	Relative content (%)				
			Control	TPJ-80	TPJ-100	TPJ-120	TPJ-140
unknown-1	$C1_0H_{14}O$	150.2	1.47 ± 0.08^{c}	1.89 ± 0.04^{b}	2.1 ± 0.13^{a}	2.04 ± 0.1^{ab}	1.91 ± 0.1^{b}
unknown-2	$C_{11}H_{18}O_2$	182.3	0.76 ± 0.03^{a}	0.79 ± 0.03^{a}	0.78 ± 0.03^{a}	0.72 ± 0.03^{a}	0.73 ± 0.06^{a}
unknown-3	$C_8H_{16}FO_2P$	194.2	1.31 ± 0.16^{a}	0.89 ± 0.03^{b}	0.66 ± 0.02^{c}	0.51 ± 0.04^{d}	0.42 ± 0.01^{d}
unknown-4	$C_8H_{14}O_2$	142.2	0.81 ± 0.06^{a}	0.69 ± 0.04^{b}	0.55 ± 0.04^{c}	0.43 ± 0.01^{d}	0.41 ± 0.03^{d}
unknown-5	$C_{10}H_{23}NS$	189.4	1.46 ± 0.03^{b}	3.25 ± 0.57^{ab}	1.76 ± 0.55^{b}	2.47 ± 0.99^{a}	4.34 ± 1.88^{a}
unknown-6	$C_{10}H_{21}NOSi$	199.4	8.32 ± 0.27^{ab}	7.76 ± 0.05^{b}	8.98 ± 0.03^{a}	8.24 ± 0.53^{ab}	8.55 ± 0.65^{a}
unknown-7	$C_{10}H_{16}O$	152.2	0.9 ± 0.02^{ab}	0.95 ± 0.09^{ab}	0.99 ± 0.06^{a}	0.88 ± 0.03^{b}	0.74 ± 0.05^{c}
unknown-8	C_7H_5NS	135.2	1.06 ± 0.06^{a}	1.02 ± 0.03^{a}	0.94 ± 0.11^{a}	0.83 ± 0.07^{b}	0.68 ± 0.03^{c}
unknown-9	$C_{10}H_{16}O$	152.2	9.08 ± 0.6^{d}	11.03 ± 0.08^{b}	11.85 ± 0.13^{a}	10.01 ± 0.44^{c}	8.53 ± 0.37^{d}
unknown-10	$C_6H_4ClNO_2$	157.6	2.01 ± 0.12^{a}	1.75 ± 0.04^{ab}	1.6 ± 0.08^{b}	1.12 ± 0.07^{c}	0.91 ± 0.27^{c}
unknown-11	$C_{10}H_{18}O$	154.3	0.79 ± 0.08^{b}	1.61 ± 0.02^{a}	1.61 ± 0.03^{a}	1.45 ± 0.02^{a}	0.92 ± 0.23^{b}
unknown-12	$C_{10}H_8$	128.2	0.79 ± 0.06^{a}	0.61 ± 0.01^{b}	0.55 ± 0.02^{b}	0.29 ± 0.03^{c}	0.15 ± 0.03^{d}
unknown-13	$C_{12}H_{24}$	168.3	0.7 ± 0.02^{a}	0.61 ± 0.01^{b}	0.55 ± 0.03^{c}	0.41 ± 0.03^{d}	0.4 ± 0.03^{d}
unknown-14	$C_{10}H_{20}O$	156.3	0.15 ± 0.12^{a}	0.19 ± 0.03^{a}	0.19 ± 0^{a}	0.17 ± 0.01^{a}	0.17 ± 0.07^{a}
unknown-15	$C_4H_{10}O_2S$	122.2	0.84 ± 0.18^{b}	1.04 ± 0.05^{b}	0.99 ± 0.15^{b}	1.68 ± 0.93^{b}	2.73 ± 0.38^{a}
unknown-16	$C_{10}H_{18}O_2$	107.3	0.67 ± 0.02^{b}	0.41 ± 0^{d}	0.42 ± 0.02^{d}	0.55 ± 0.09^{c}	1.06 ± 0.05^{a}
unknown-17	$C_7H_{14}O_2$	130.2	0.97 ± 0.09^{bc}	0.82 ± 0.02^{c}	1.03 ± 0.02^{bc}	1.23 ± 0.09^{b}	1.73 ± 0.3^{a}

Supplementary Table S2 Color parameters of sponge cakes prepared with different baking temperatures

Measurement site	Sample	L*	a*	b*	△E	Appearance
crust	PFC-120 °C	62.48 ± 0.68^{a}	2.63 ± 1.01^{d}	36.84 ± 0.76^{c}	—	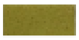
	PFC-130 °C	63.62 ± 0.99^{a}	4.5 ± 0.63^{c}	38.61 ± 0.63^{b}	2.68 ± 0.65^{c}	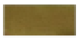
	PFC-140 °C	63.15 ± 0.45^{a}	7.14 ± 0.29^{b}	40.76 ± 0.60^{a}	5.71 ± 0.21^{b}	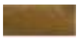
	PFC-150 °C	52.85 ± 1.58^{b}	11.39 ± 0.89^{a}	41.99 ± 0.66^{a}	14.08 ± 0.70^{a}	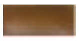
crumb	PFC-120 °C	56.59 ± 0.99^{a}	-5.32 ± 0.66^{a}	42.12 ± 0.72^{a}	—	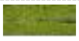
	PFC-130 °C	56.09 ± 0.62^{a}	-7.56 ± 0.40^{b}	40.73 ± 0.51^{ab}	3.09 ± 0.51^{b}	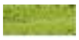
	PFC-140 °C	56.18 ± 1.62^{a}	-8.83 ± 0.34^{c}	39.87 ± 0.57^{b}	4.61 ± 1.02^{ab}	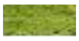
	PFC-150 °C	56.77 ± 0.89^{a}	-10.12 ± 0.62^{d}	39.96 ± 1.51^{b}	5.85 ± 0.96^{a}	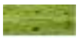

[a]"Control" represents the fresh pandan leaf juice without treatment; "PFC-120 °C", "PFC-130 °C", "PFC-140 °C" and "PFC-150 °C" denotes pandan-flavored cakes baked under 120 °C, 130 °C and 140 °C, respectively; In the same row with different letters denotes significant difference at the level of 0.05.

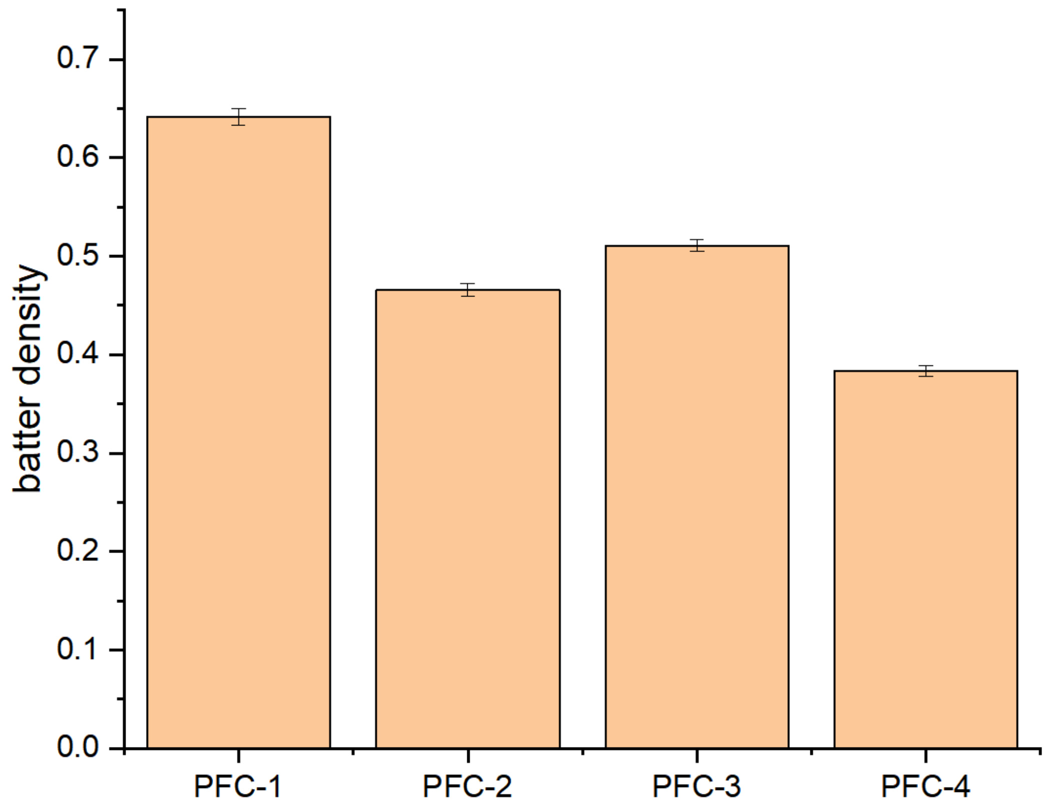

Supplementary Figure S1. The batter density of Pandan-flavored sponge cake samples supplemented with 0%, 10.3%, 13.3%, 16.1% and 18.8% of Pandan leaf juice, respectively
